# Supplementary material for: Content-rich biological network constructed by mining PubMed abstracts
Source: BMC Bioinformatics. 2004 Oct 8;5:147. doi: 10.1186/1471-2105-5-147 (PMC528731; doi:10.1186/1471-2105-5-147)
Supplement: Additional File 2 — The original results of the above study (non-essential files are deleted to keep the file size under the limit set by BMC bioinformatics). [file 1471-2105-5-147-S2.bz2 › chilibotAdditionalFile2/dip05/59ID8414517E214/html/RBBP2_RB1.html]

 


 **RBBP2** and **RB1** 
  
Found 11 abstracts in PubMed, retrieved 05.  
 

 What does Google say? 
 PDF only 
| .edu only 

---

**Interactive relationship** (e.g. stimulation, inhibition, etc)

**Neutral relationship**- Retinoblastoma  [ **RB1** ]  binding protein 2  [ **RBBP2** ]  Rbp2 was originally identified as a retinoblastoma  [ **RB1** ]  protein RB pocket domain binding protein.  Ref: 11358960 J Biol Chem, 2001

**Non-interactive relationship** (e.g. studied together, co-existance, homology, etc.)

- The protein encoded by the little imaginal discs gene is the Drosophila homologue of human retinoblastoma  [ **RB1** ]  binding protein 2  [ **RBBP2** ] .  Ref: 11014813 Genetics, 2000
- these genes encode very diverse proteins, including signaling molecules such as BMP 4, the enzyme superoxide dismutase, the cell adhesion molecule cadherin 6, proteins involved in gene transcription such as HMG 1 and SAP18, homeodomain containing proteins Gbx 2 and Evx 2, and cell cycle regulatory proteins such as the retinoblastoma  [ **RB1** ]  binding protein 2  [ **RBBP2** ] .  Ref: 10942599 Exp Cell Res, 2000
- A screen for new trithorax group genes identified little imaginal discs, the Drosophila melanogaster homologue of human retinoblastoma  [ **RB1** ]  binding protein 2  [ **RBBP2** ] .  Ref: 11014813 Genetics, 2000
- A protein with similarity to the human retinoblastoma  [ **RB1** ]  binding protein 2  [ **RBBP2** ]  acts specifically as a repressor for genes regulated by the b mating type locus in Ustilago maydis.  Ref: 11029697 Mol Microbiol, 2000
- In a screen for components of the b dependent regulatory cascade we have isolated Rum1 regulator U. maydis 1, a protein with similarities to the human retinoblastoma  [ **RB1** ]  binding protein 2  [ **RBBP2** ] .  Ref: 11029697 Mol Microbiol, 2000
- These include human hairless, mutated in individuals with alopecia universalis, retinoblastoma  [ **RB1** ]  binding protein 2  [ **RBBP2** ]  and several putative chromatin associated proteins.  Ref: 11165500 Trends Biochem Sci, 2001
- Retinoblastoma  [ **RB1** ]  binding protein 2  [ **RBBP2** ]  Rbp2 potentiates nuclear hormone receptor mediated transcription.  Ref: 11358960 J Biol Chem, 2001
